# Cervical Stimulation in the Treatment of Children with Lymphedema of All Four Extremities: A Case Report and Literature Review

**DOI:** 10.1155/2017/9724524

**Published:** 2017-02-28

**Authors:** Livia Maria Pereira de Godoy, Paula Pereira de Godoy Capeletto, José Maria Pereira de Godoy, Maria de Fátima Guerreiro Godoy

**Affiliations:** ^1^Clínica Godoy Research Group, Lusiadas University Center (UNILUS), Santos, SP, Brazil; ^2^Clínica Godoy Research Group, Votuporanga Medicine School, Votuporanga, SP, Brazil; ^3^Cardiovascular Surgery Department, The Medicine School in São José do Rio Preto (FAMERP) and CNPq (National Council for Research and Development), São José do Rio Preto, SP, Brazil; ^4^Post-Graduation Stricto Sensu Course, The Medicine School in São José do Rio Preto (FAMERP) and Clínica Godoy Research Group, São José do Rio Preto, SP, Brazil

## Abstract

*Aim*. The aim of this study is to report on the use of cervical stimulation as monotherapy to reduce swelling and normalize the size of limbs in two children with lymphedema of all four extremities.* Case Presentation*. One child also had hemifacial edema. In both cases, the mothers were trained to perform cervical stimulation under professional supervision. The cases of two girls, one of eight months and the other of six months, with primary congenital lymphedema are described.* Outcome*. After clinical diagnosis, the patients started treatment with cervical stimulation three times per week. The mothers were trained in cervical stimulation and, when the therapy team was confident about the mothers' ability to perform the technique, the children began to be treated at home. The Godoy & Godoy cervical stimulation technique consists of around 20 to 30 light stroking movements per minute in the cervical region which stimulate the lymphatics. Perimetric measurements were made of the feet, legs, and the hands. Only two points (3 and 6 cm) along the dorsum of the feet and hands and points at 5 cm intervals up the legs starting at the ankle were considered. Today, the children are 5 and 6 years of age, without edema and with a normal life, without limitations, except with respect to precautions against injuries to the limbs and against infections particularly erysipelas.* Conclusion*. Cervical Lymphatic Therapy as monotherapy is an option in the treatment of primary congenital lymphedema.

## 1. Introduction

Lymphedema is defined as the abnormal accumulation of macromolecules in the interstitial space which, in turn, leads to an accumulation of fluids due to a failure in the formation and drainage of lymph as a result of congenital or acquired lesions of the lymphatic system [1]. Lymphedema in children is rare; the prevalence is around 1.15/100.000 in under 20 year olds [2]. One study of 312 patients under 36 years old with primary lymphedema identified ten individuals who had had the disease at birth. A frequency of 1/6000 live births has been reported with a ratio of one male to every three females [3]. In respect to congenital lymphedema, there is variability in signs and symptoms between families and the age at onset varies; there are more than four known genetically distinct conditions and mutations in three genes have been discovered in families with lymphedema [4].

The diagnosis of congenital lymphedema is still a challenge. Primary lymphedema is classified into two subgroups: idiopathic and familial (hereditary) [5]. In general, it is recommended that when peripheral lymphedema of undetermined etiology and with dimorphisms is found, an evaluation of associated syndromes should be carried out [6]. The recommended treatment of lymphedema is an association of therapies such as Manual and Mechanical Lymphatic Therapy, compression mechanisms (stockings and bandages), myolymphokinetic activities, and exercises and care with hygiene [7].

In recent years, new techniques of lymph drainage have been developed. One, called Cervical Lymphatic Therapy (previously called cervical stimulation), allows the reduction and control of swelling [8]. One advantage of this technique is that mothers can be trained to perform the therapy, thereby making them partially responsible for the treatment of their child with lymphedema. Although constant training and supervision of the mothers are essential, this strategy gives a little more independence to the family. However, there is lack of studies reporting the development of different forms of treatment used in children.

The aim of this study is to report on the use of cervical stimulation as monotherapy to reduce swelling and normalize limb size in two children with lymphedema of the four extremities. One child also had hemifacial edema. In both cases, the mothers were trained to perform cervical stimulation under professional supervision.

## 2. Case Report 1

The case of a female child is reported who was evaluated at the age of eight months. The mother reported that the child had had edema since birth, mainly of the back of the hands and feet, which she observed when changing the baby. The family consulted with a pediatrician who accompanied the child monthly and asked to wait for a few months to follow the evolution of the child. At eight months of age, the child was referred to an orthopedic doctor who did not diagnose any problem related to his area and referred the patient to a vascular surgeon. In the initial physical evaluation, the child had edema of the dorsum of the feet and of the hands including the fingers and the Stemmer sign was positive. The mother did not report any kind of trauma, skin lesion, or infection of the fingers. According to the mother, the gestation was normal when she had monthly consultations with her doctor, childbirth was natural without any complications, and there is no family history of lymphedema. Clinical diagnosis of primary congenital lymphedema was confirmed. After diagnosis, the mother was advised about the disease and the need for treatment and basic day-to-day care.

The child began treatment with cervical stimulation [8] three times per week for three months with total reduction of edema. The Godoy & Godoy Cervical Lymphatic Therapy consists of around 20 to 30 light stroking movements per minute in the cervical region to stimulate the lymphatics. Perimetric measurements were made of the feet, legs, and the backs of the hands. Only two points (3 and 6 cm) along the dorsum of the feet and hands and points at 5 cm intervals up the legs starting at the ankle were considered. Cervical Lymphatic Therapy was explained to the mother during the 15-minute treatment sessions and the mother was taught how to perform the technique. After training, the mother started to treat her child in the clinic under the supervision of a therapist. When the medical team was confident about the mother's ability to perform the technique, she started treating her child at home with weekly control consultations in the clinic. Currently, the patient is five years old, without edema and has a normal life, without limitations except related to preventing injuries and infections in particular erysipelas.

## 3. Case Report 2

**Figure 1:**
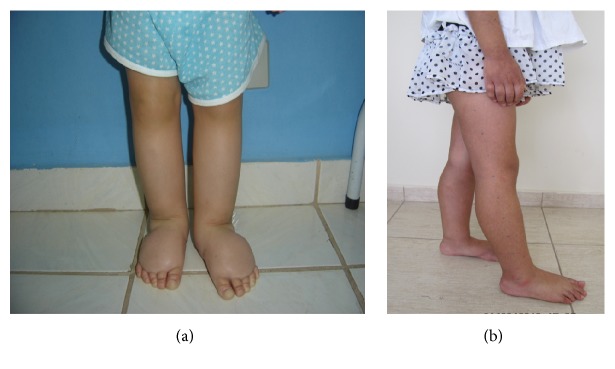
Images of the child (a) before treatment and after treatment (b).

The case of a six-month-old female child is reported with edema of the legs, hands, and hemiface. She was referred by her pediatrician with a history of edema from birth. The pediatrician also requested evaluations by a pulmonologist and an ear-nose-throat specialist because of frequent episodes of pneumonia, apparent fatigue when playing, reflux, and restless sleep. Deviated nasal septum was diagnosed with other complementary examinations (cardiac, pneumology, and magnetic resonance) being normal. The vascular evaluation detected hard edema of the dorsum of feet, the hands, fingers, and right hemiface. The family reported that the child had repetitive nail infections making clipping difficult. The child at that time had not started to walk or sit and had not spoken a single word. The mother reported a normal gestation accompanied by the doctor on a monthly basis. Delivery was by C-section without any complication. There was no family history of lymphedema. Clinical diagnosis of primary congenital lymphedema was confirmed. After diagnosis, the mother was advised about the disease and about the need for treatment and basic day-to-day care to improve the edema and control the size of the limbs within normality. She was also advised about the importance of stimulating the child to walk and talk, given guidance regarding skin injuries and hygiene and the appropriate use of footwear to improve the control of the lymphedema. The child began treatment with 15-minute therapeutic sessions of cervical stimulation [8] three times per week for three months. The Godoy & Godoy Cervical Lymphatic Therapy consists of around 20 to 30 light stroking movements per minute in the cervical region to stimulate the lymphatics. Perimetric measurements were made of the feet, legs, and the backs of the hands. Only two points (3 and 6 cm) along the dorsum of the feet and hands and points at 5 cm intervals up the legs starting at the ankle were considered. Cervical Lymphatic Therapy was explained to the mother during the treatment sessions and the mother was taught how to perform the technique at home. Currently, the patient is six years old and has a normal quality of life, without edema that had normalized after six months of treatment (Figures 1(a) and 1(b)). There are no limitations in her daily routine due to the lymphedema. The need for constant care to prevent injuries and infections, especially erysipelas, was carefully explained to the mother.

## 4. Discussion

This study describes a new option in the treatment of primary congenital lymphedema using Cervical Lymphatic Therapy (cervical stimulation) as monotherapy to reduce edema; prophylactic care against injuries and infection is required always. These children suffer from lymphedema of the four extremities and one of the right hemiface, and so the involvement of the lymphatic system is significant. The child with edema of the hemiface started with an increase in the size of one leg, but without clinical features suggestive of Klippel-Trenaunay syndrome. The possibility of this being a syndrome is still being investigated.

One important characteristic of this approach is that the mother can be trained to perform the technique safely. This is important for treatment adherence as it is difficult to treat children during some phases of their lives as they do not always sit still and stay quiet and so it is not always easy to perform treatment correctly. Thus, training the mother to perform this technique while the child is asleep is a good alternative. This technique can be learnt by thousands of mothers around the world, and may benefit the health and quality of life of thousands of children who are today without adequate treatment.

Currently, there are about 40 children in the service being treated using this technique and some with other associated therapies. However, these associations must have synergistic effect to reduce the edema, for example, the association of a compression stocking made of a cotton-polyester fabric (grosgrain) [9]. The two cases presented in this study are the only ones with lymphedema of all four extremities.

Another aspect to be considered is that these children have a normal life, without compression garments or anything else that could make them feel uncomfortable and so their quality of life is within the parameters expected for a normal child. The necessary precautions are related to injuries and hygiene of the extremities in order to prevent any infection. Hence, the life of the children is practically normal; they are permitted to participate in all activities, including ballet, swimming, and dance. However, monitoring is very important to identify possible worsening of the edema.

Children are referred to the clinic from different regions of Brazil and from other countries, but often without being treated in any way. In these cases, there is a need to adapt therapy to the reality of each patient and the situations in which they find themselves. The association of the grosgrain compression garment provides a synergistic effect to reduce edema, but it is uncomfortable for many children to use. Therefore, possible cervical stimulation is used as monotherapy. Manual and Mechanical Lymphatic Therapies are other therapeutic options, depending on the case in particular the age of the child.

## 5. Conclusion

Cervical lymphatic therapy as monotherapy is an option in the treatment of primary congenital lymphedema.
